# Novel insights into the cognitive, emotional, and experiential dimensions of stakeholder acceptance of wildlife management

**DOI:** 10.1038/s41598-024-80661-2

**Published:** 2024-11-27

**Authors:** Louise Eriksson, Maria Johansson, Johan Månsson, Camilla Sandström, Niklas Liljebäck, Johan Elmberg

**Affiliations:** 1 Department of Geography, Umeå University, 901 87 Umeå, Sweden; 2 Environmental Psychology, Department of Architecture and Built Environment, Lund University, 221 00 Lund, Sweden; 3 Grimsö Wildlife Research Station, Department of Ecology, Swedish University of Agricultural Sciences, 730 91 Riddarhyttan, Sweden; 4 Department of Political Science, Umeå University, 901 87 Umeå, Sweden; 5 Department of Environmental Science and Bioscience, Kristianstad University, 291 88 Kristianstad, Sweden

**Keywords:** *Anser*, *Branta*, Geese, Damage reduction, Multilevel management, The cognitive and emotional hierarchy model, Psychology, Environmental social sciences

## Abstract

Understanding wildlife stakeholders is vital in mitigating the risk for inertia in the implementation of management and illegal activities, e.g., poaching. We used a unique set of questionnaire data of stakeholders in Sweden (*n* = 8728) comprising birdwatchers, hunters, farmers, and the general public, to analyze evaluations of geese, beliefs about goose management at multiple levels, and acceptance of management tools. Also, a hierarchical model incorporating prior wildlife experience and integrating cognitions and emotions for acceptance was tested. The overall attitude towards geese was positive, but ecosystem disservices caused by large populations were acknowledged. The general public and birdwatchers displayed trust in the national, regional, and local levels. In contrast, farmers and hunters trusted mainly the local level. Broad stakeholder acceptance was found for several non-lethal tools for damage reduction and more generally a goose conservation approach including e.g., hunting free zones, but also for hunting during open hunting season. We found support for the proposed model in relation to acceptance of both the conservation and the lethal approach, thereby advancing theory integration. Results suggest that while goose conservation is widely accepted, problems caused by geese and distrust in the higher management levels among some stakeholder groups need to be addressed.

**Supplementary Information:**

The online version contains supplementary material available at 10.1038/s41598-024-80661-2.

## Introduction

Stakeholders involved in wildlife management can represent diverse interests and have varied perceptions of ecosystem services and disservices of wildlife. They may endorse the conservation of species and their habitat, the utilization of wildlife populations or individuals as resources (e.g., hunting and tourism), the extraction of products, or cultivation of habitats shared with wildlife^1–3^. The last decades, wildlife management is increasingly conducted in a social-ecological frame with an emphasis on collaboration. Therefore, involvement from diverse stakeholder groups is needed at different levels in the system, including local, regional, national, and international levels^4,5^. There is a growing realization that management, including conservation, needs to align with stakeholder representatives involved in management, but also with stakeholder groups in general (e.g., local residents, farmers, hunters, and members in environmental organizations) as well as the general public^6–8^. Insight of stakeholders’ wildlife management acceptance is warranted to enable pro-active efforts directed at reducing the risk of polarization between stakeholder groups, inertia in the implementation of management, distrust, and illegal activities, e.g., poaching^7–9^.

Earlier studies on acceptance of wildlife management have relied heavily on small and non-random samples and have often been limited to certain stakeholder groups (e.g., hunters or conservationists, but see^10^). Moreover, the focus has primarily been on either reducing damages through e.g., lethal control, or conservation. By addressing these limitations with appropriate sampling methods and a broad approach, conclusions will be more reliable and thereby ensuring accurate advice for management. Psychological models can improve the understanding of stakeholders and their experiences, and also how they think and feel, to explain attitudes expressed and behavioral reactions towards management. Cognitive variables, such as value orientations and beliefs, and emotions e.g., fear, are confirmed predictors of acceptance of wildlife management^11–15^. However, to learn from research of stakeholders’ management acceptance across species and contexts there is a need for consolidation by integrating theory and research.

The present study explores acceptance of management tools, including conservation interventions, to provide insights for the emerging multi-level management of geese in Europe, where increasing populations of some species (but decline of others) create conservation conflicts and thereby add challenges to flyway management^5,16,17^. We used large stakeholder samples and a broad coverage of stakeholder groups (birdwatchers, hunters, farmers, general public) in Sweden, to analyze beliefs about multiple levels of management (from local to European) and acceptance of tools used in goose management. Finally, the study enhances theory development by emphasizing experiential processes and integrating emotions into the cognitive hierarchy model^18^ by drawing on the tripartite model of attitude^19^.

## Background

Previous studies have examined stakeholders’ acceptance of different tools used in wildlife management^2,3,8,14,20–23^. In general, there is more support for non-lethal tools (e.g., the use of deterrents, fencing) compared to lethal ones and even when lethal tools are supported, stakeholders often emphasize ethical considerations^1,15,23^ (but see^24^). Yet, acceptance levels are highly dependent on stakeholder group, with conservationists often displaying a low acceptance for lethal tools even for abundant species^8^. The acceptance for certain strategies may also depend on target species^25^. For example, conservationists may support lethal approaches for alien and invasive species^26^. Acceptance is further contingent on the extent to which wildlife is related to apparent disservices as threatening, causing crop damage, or transferring diseases^8,12,27^. Moreover, acceptance may be site specific and context dependent, e.g., lower acceptance of carnivores in rural areas, most pronounced when carnivores are present and in specific stakeholder groups, such as farmers^28,29^ (but see^30^). Psychological factors, such as wildlife value orientations (WVOs), beliefs, norms, emotions, attitudes, identity, and management beliefs have been examined in relation to acceptance^15,20,31–34^. With regard to wildlife management, it is important to consider stakeholders’ evaluation of wildlife per se (e.g., attitude towards wildlife and acceptance capacity reflecting perceptions of whether the wildlife population should decrease or increase^35^), stakeholder beliefs about management tools, as well as stakeholders’ relations with management agencies, in terms of ascribed responsibility, trust, and participation^36,37^. The few studies examining acceptance of both lethal and non-lethal tools suggest that e.g., WVOs and emotions are not consistent predictors of acceptance of different management tools across contexts^13,14,38^. To understand whether determinants of acceptance depend on the characteristics of the management approach (e.g., lethal versus non-lethal), research needs to comprise a broad coverage of stakeholders using a coherent theoretical approach when examining acceptance of diverse sets of management tools.

### The cognitive and emotional hierarchy model

Acceptance of wildlife management may be conceptualized in terms of an attitude, i.e., an evaluation ranging from more positive to negative^19^. There are several theoretical models that outline the psychological foundation of attitudes. One of them is the cognitive hierarchy model, which states that hierarchically ordered values and thoughts on a topic constitute the basis of attitudes and subsequently behaviors^18,39^. Nevertheless, evidence is mounting for the importance of emotions for acceptance^40^. For example, Doney et al.^11^ outlined a conceptual framework where WVOs together with emotions evoked by wildlife are important for acceptance of management and Slagle et al.^12^ proposed a model in which fear and beliefs influenced intentions to support large carnivore conservation. However, the experiential foundation of beliefs and emotions, and a more complete integration of emotions into the cognitive hierarchy model are still lacking. Progress towards theory integration may be made by drawing on the tripartite model of attitudes^19^ positing that cognitive, emotional and prior behavioral processes are important to the foundation of attitudes. Thus, in addition to general cognitive factors, such as basic values and WVOs, individual and situationally based wildlife experiences become important for the formation of attitudes^35^.

**Fig. 1:**
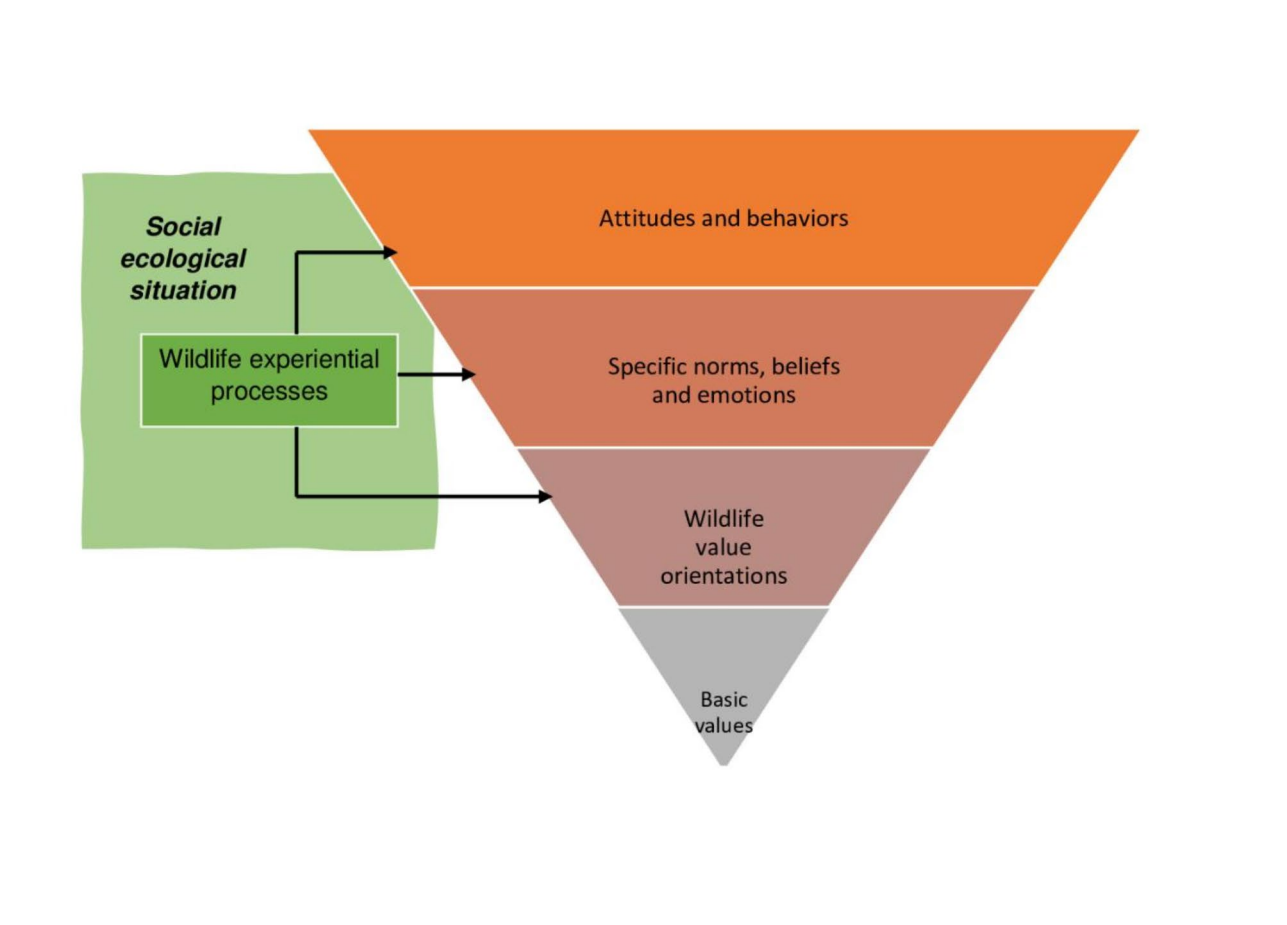
Cognitive and emotional hierarchy (CEH) model (based on the cognitive hierarchy model in Fulton et al.^18^ and the tripartite model of attitudes^19^).

The cognitive hierarchy model stipulates that basic values, transcending situations, and more domain-specific general beliefs, often labelled value orientations (e.g., WVOs), are considered few and slow to change. In contrast, specific beliefs, i.e., thoughts about the object^19^, including normative beliefs reflecting personal standards for actions^41^ and personal moral norms (a sense of obligation to act)^33^ are numerous and can change more easily. A utilitarian orientation also labelled domination, where wildlife is seen as to be used for human needs, has been found to be associated with acceptance of lethal tools. On the other hand, mutualism reflecting an egalitarian orientation, where wildlife, too, is considered to have rights, is associated with higher acceptance for non-lethal tools and less for lethal tools^10,13,22^. There is further support that personal norms, specific beliefs, and attitudes towards wildlife are even more strongly associated with evaluations of wildlife management than more general cognitions, such as WVOs^42,43^ (but see^11^). The account of how beliefs are linked to the attitude object through propositional connections as part of a cognitive network^44^ is in line with the cognitive hierarchy model. However, the tripartite model of attitudes also suggests that emotions are connected to attitudes through evaluative conditioning, i.e., an objects’ pairing with a stimulus that is positively or negatively charged^45^. Other accounts suggest that constantly occurring appraisals of an event (e.g., an external stimuli) determine the elicitation of emotions^46^, thereby depicting how experiences of an event, interpretative processes, and emotions are connected. Furthermore, the tripartite model of attitudes suggests that the latter can be inferred based on the mere consideration of past behaviors (so called self-perception processes), often when the topic has not been given much thought^47^. In the wildlife management context, nature experiences when involved in different activities, such as birdwatching or hunting, are likely to be part of the attitude formation process. Taken together, there are several arguments for expanding the cognitive hierarchy model by integrating additional psychological processes likely to shape stakeholder acceptance. We propose an integrated cognitive and emotional hierarchy (CEH) model, highlighting that the social ecological situation in which experiential processes evoke thoughts and emotions are part of the foundation of acceptance for wildlife management (Fig. 1). Similar to the cognitive hierarchy model, basic values and WVOs are general cognitive factors relevant for attitudes, via specific beliefs and norms. In addition, the CEH model suggests that emotions are, similar to specific beliefs and norms, important for attitudes. Finally, it connects experiences of wildlife as part of the social ecological context to the psychological processes involved in attitude formation. The different levels in the models outline key distinctions but additional hierarchical ordering within the top levels (e.g., attitudes and behaviors) is possible. This model may explain why more specific cognitions and attitudes, including acceptance of wildlife management, may change over time when WVOs remain relatively stable.

### Study objectives

This study examines WVOs, beliefs, emotions, and acceptance of goose management across a range of stakeholder groups in Sweden, including birdwatchers, hunters, farmers and with the general public used as a baseline for comparisons. The study has three overall objectives.

1. To examine evaluations of geese in terms of attitude and acceptance capacity as well as stakeholders’ beliefs about responsibility and trust in the different goose management levels (local to EU).
2. To analyze acceptance of management tools in the different stakeholder groups with the aim to explore levels of acceptance as well as differences and similarities within and between groups. Due to differences in WVOs and experiences, we expect birdwatchers to display higher acceptance for goose conservation than the other groups. Hunters and farmers are expected to display higher acceptance for management prioritizing human utilization of land and wildlife than do birdwatchers.
3. Drawing on the CEH model outlined above, we examine the importance of WVOs, beliefs about geese in terms of ecosystem services and disservices, and the emotions geese evoke for acceptance of core management approaches, also considering the role of experiential variables (i.e., outdoor activities) and a set of control variables. We expect WVOs to be predictors of acceptance, but goose specific beliefs and emotions to have even stronger effects. In addition, we expect significant associations between experiences with outdoor activities and acceptance.

## Results

The share of women was much lower among birdwatchers, hunters, and farmers than in the general public (Table 1). Birdwatchers had the highest mean age, 68 years, while the remaining groups were on average about 50 years old. Birdwatchers had the highest formal educational level, followed by the general public, hunters, and finally, farmers. The groups displayed expected differences in experience of outdoor activities with the general public being more similar to birdwatchers regarding hunting, to hunters regarding visits in natural areas, and to farmers regarding birdwatching.

**Table 1:** Sample characteristics.

	The general public	Birdwatchers	Hunters	Farmers	*P* value
Study year	2018	2021	2022	2019	
Net sample	2973	15,700	4930	2973	
Response frequency	30%	32%	36%	36%	
N_response_	898	5010	1753	1067	
Gender (women)	51%	26%	10%	19%	0.001
Age	53 years (SD = 16)^b^	68 years (SD = 12)^d^	50 years (SD = 12)^a^	55 years (SD = 9)^c^	0.001
Education (university degree)	49%	67%	40%	33%	0.000
Visiting natural areas^1^	79%	92%	86%	47%	0.001
Birdwatching^1^	20%	96%	44%	23%	0.001
Hunting^1,2^	5%	7%	94%	36%	0.001

P values indicate an overall significant Chi square test, except for age where the P value indicate a significant univariate ANOVA and means having the same superscript letter did not differ at *p* < 0.05 (multiple comparisons with Bonferroni correction).

^1^At least one or a few times a year.

^2^. The general public was asked about hunting of wild birds and big game. Birdwatchers, hunters, and farmers was asked about hunting of wild birds, small game, and big game.

### Evaluations of geese and beliefs about goose management

**Fig. 2:**
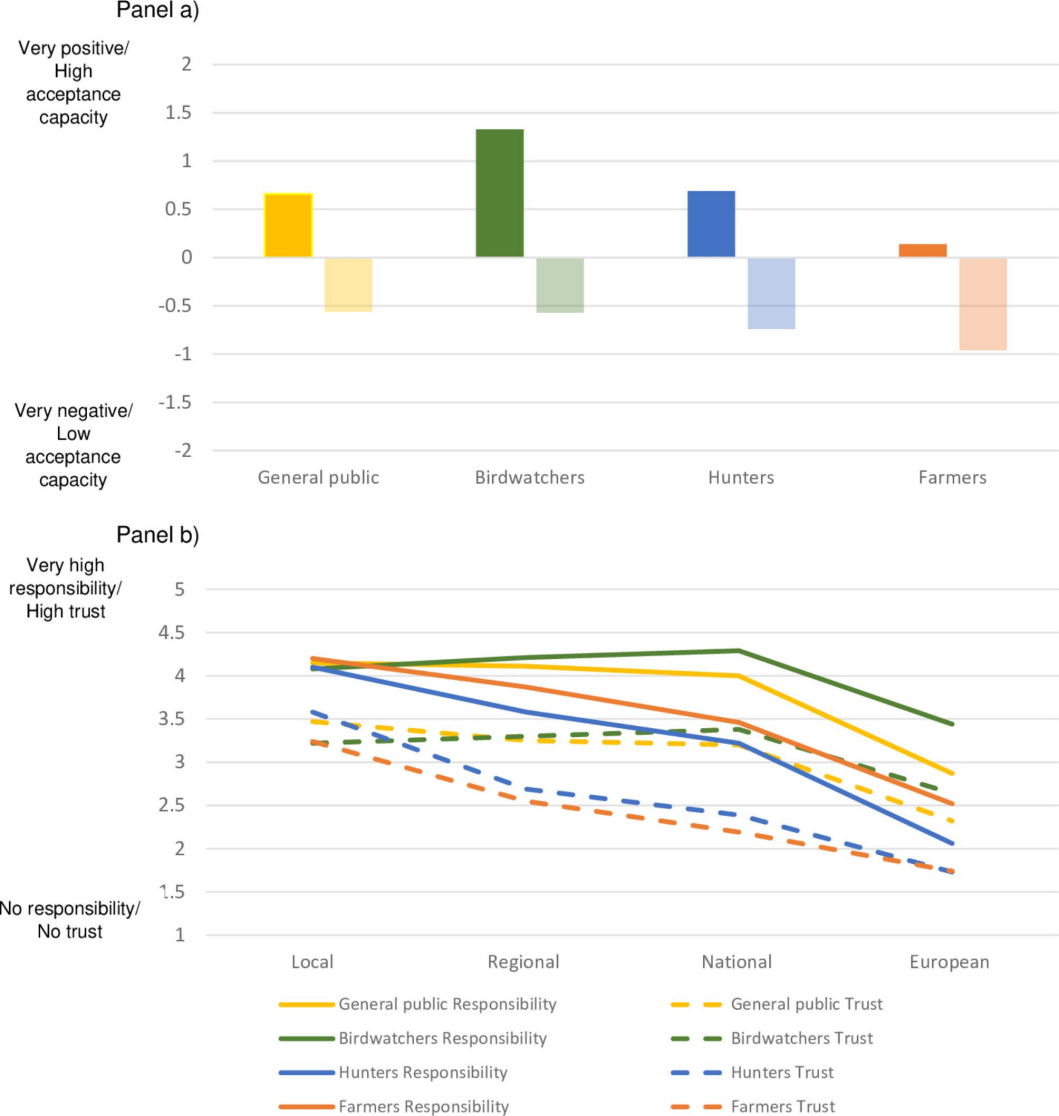
Evaluations of geese (attitude (deeper color) and acceptance capacity (paler color)) (panel a), ascribed responsibility for goose management at the local to European level (solid line) and trust in the different levels for managing geese (dashed line) (panel b) in the stakeholder groups.

The stakeholder groups displayed an overall positive or neutral attitude towards geese, but low acceptance capacity (top panel, Fig. 2). Whereas birdwatchers had the most positive attitude towards geese, farmers were close to neutral. Farmers had the lowest acceptance capacity of geese, followed by hunters, with a slightly higher acceptance capacity among birdwatchers and the general public. There was a greater difference in attitude than in acceptance capacity between the stakeholder groups (*Partial eta*^*2*^ = 0.25 and 0.03, respectively).

All groups ascribed a high level of responsibility to the local level and lower levels of responsibility to the EU level (bottom panel, Fig. 2). The difference in ascribed responsibility between management levels was particularly evident among hunters and farmers. Significant differences between all groups were confirmed only for the national and the EU levels (*Partial eta*^*2*^ = 0.00, 0.05, 0.14, and 0.14 respectively). While the general public and the birdwatchers displayed a relatively high trust in the local, regional, and national levels, farmers and hunters mainly trusted the local level. The four groups were most similar regarding trust in the local level (only birdwatchers and farmers differed significantly, *p* = 0.016), followed by trust in the regional level (where all groups differed *p* = 0.001, except the general public and birdwatchers). The differences in trust were more pronounced among the stakeholder groups for the higher management levels (*Partial eta*^*2*^ = 0.02, 0.07, 0.16, and 0.12, respectively).

### Acceptance of goose management tools

**Fig. 3:**
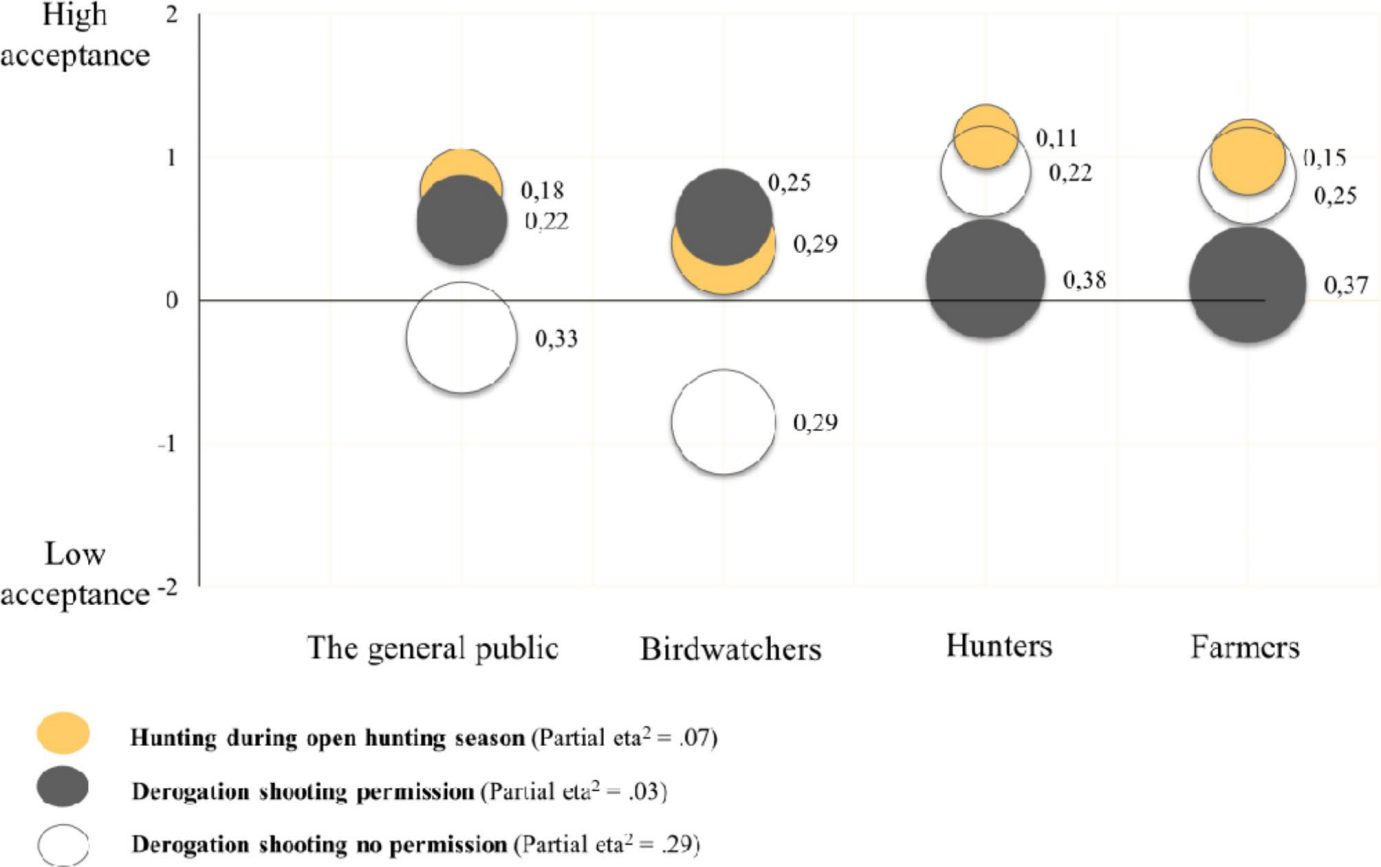
Acceptance of hunting during open season and two types of derogation shooting; (1) when a permit from the County Administrative Board is needed and (2) when it is not needed, in the stakeholder groups as an illustration of acceptance for different hunting tools (within group differences, PCI_2,_ next to each bubble, and between group differences, Partial eta^2^).

Acceptance for the goose management tools is shown in Table 2, with visual illustrations of acceptance for hunting during open season and two types of derogation shooting (outside open season): (1) when a permit from the CAB is needed, and (2) when it is not needed, in Fig. 3. The remaining illustrations are displayed in Appendix Fig. 1A. The stakeholder groups displayed an overall acceptance for several of the goose management tools, including financial compensation to farmers for damages, visual scaring, some forms of hunting (especially during open season), and a tool kit combining derogation shooting, scaring, and diversionary fields. As a single tool, diversionary fields were accepted by all groups, but there was substantial between-group variation with high acceptance among birdwatchers and average among farmers. For derogation shooting that requires a permission, there was little between-group variation, but some within-group variation among hunters and farmers, indicative of sub-groups with a lower acceptance. The mean values indicated an overall acceptance for use of distasteful crops, scaring to diversionary fields, low fences, and auditory scaring. However, the measure of within-group variation (PCI_2_) reveals that at least half of the hunters and farmers display a lower acceptance for these tools, and at least half of the birdwatchers displayed a lower acceptance for auditory scaring. For lethal tools in terms of capture/hunting of goslings and pricking of eggs, acceptance was overall low, despite medium to large differences between the groups. For two of the tools, hunting free zones and derogation shooting when no permission is required, the stakeholder groups displayed large disagreement, with strong support for the first-mentioned tool among birdwatchers and strong support for the last-mentioned tool among hunters and farmers. The confirmatory factor analysis of acceptance of seven tools (with medium to large group differences) revealed a lethal versus a conservation approach (eigenvalues 2.97, 1.18, respectively) explaining 59% of the variance (reliabilities are displayed in Appendix Table 1A). All expected differences among stakeholder groups were evident. However, it is noteworthy that all groups generally accepted the conservation approach, and only birdwatchers displayed a markedly low acceptance for the lethal approach.

**Table 2:** Acceptance of goose management tools (means and standard deviations), within-group differences (PCI_2_), and between-group differences (partial eta^2^) in the stakeholder groups.

	The general public		Birdwatchers		Hunters		Farmers		Effect size
	M (SD)	PCI_2_	M (SD)	PCI_2_	M (SD)	PCI_2_	M (SD)	PCI_2_	Partial eta^2^
Overall acceptance									
C: Diversionary fields^1^	0.93 (0.91)^b^	0.10	1.28 (0.81)^a^	0.08	0.51 (1.04)^c^	0.19	0.24 (1.19)^d^	0.28	**0.16**
N-L: Financial compensation to farmers	0.62 (1.03)^b^	0.15	1.05 (0.87)^a^	0.10	0.72 (1.09)^b^	0.18	1.12 (1.00)^a^	0.12	0.03
L: Hunting during open hunting season^2^	0.77 (1.10)^c^	0.18	0.40 (1.22)^d^	0.29	1.14 (0.96)^a^	0.11	1.00 (1.02)^b^	0.15	**0.07**
N-L: Visual scaring	0.96 (0.96)^a^	0.11	0.47 (1.08)^b^	0.22	0.34 (1.10)^c^	0.21	0.26 (1.14)^c^	0.24	0.03
C: Larger zones where geese are allowed to graze, and farmers are compensated^1^	0.24 (1.04)^b^	0.16	0.69 (0.99)^a^	0.15	0.06 (1.11)^c^	0.20	0.06 (1.17)^c^	0.24	**0.07**
L: Derogation shooting under permission	0.53 (1.15)^a^	0.23	0.58 (1.12)^a^	0.25	0.15 (1.36)^b^	0.38	0.11 (1.35)^b^	0.37	0.03
TK: Derogation shooting, scaring and diversionary fields	0.52 (1.12)^a^	0.19	0.18 (1.13)^b^	0.25	0.54 (1.04)^a^	0.16	0.47 (1.09)^a^	0.19	0.02
Intermediate acceptance									
N-L: Distasteful crops	0.41 (1.12)^a^	0.19	0.41 (1.13)^a^	0.23	0.06 (1.17)^b^	0.23	-0.17 (1.19)^c^	0.23	0.03
TK: Scaring to diversionary fields	0.37 (1.08)^a^	0.19	0.29 (1.11)^a^	0.24	-0.08 (1.12)^b^	0.21	-0.12 (1.12)^b^	0.21	0.03
N-L: Low fences	0.32 (1.18)^a^	0.25	0.03 (1.23)^b^	0.31	-0.19 (1.18)^c^	0.26	-0.30 (1.18)^c^	0.24	0.02
N-L: Auditory scaring	0.14 (1.19)^a^	0.26	-0.18 (1.17)^b^	0.28	-0.24 (1.15)^b^	0.22	-0.20 (1.15)^b^	0.23	0.01
Low acceptance									
L: Pricking of eggs^2^	-0.30 (1.18)^b^	0.21	-0.98 (1.17)^c^	0.25	-0.37 (1.25)^b^	0.26	0.00 (1.29)^a^	0.28	0.09
L: Capture/hunting of goslings and adult geese when flightless^2^	-0.54 (1.19)^b^	0.23	-1.18 (1.06)^c^	0.19	-0.56 (1.16)^b^	0.23	-0.10 (1.29)^a^	0.31	**0.11**
Disagreement									
C: Hunting free zones^1^	0.38 (1.20)^b^	0.23	0.90 (1.05)^a^	0.17	-0.40 (1.19)^c^	0.24	-0.42 (1.22)^c^	0.25	**0.22**
L: Derogation shooting no permission^2^	-0.26 (1.31)^b^	0.33	-0.85 (1.22)^c^	0.29	0.90 (1.14)^a^	0.22	0.87 (1.20)^a^	0.25	**0.29**
Management approaches									
Conservation approach (3)	0.52 (0.78)^b^	-	0.95 (0.73)^a^	-	0.06 (0.82)^c^	-	-0.03 (0.94)^d^	-	**0.22**
Lethal approach (4)	-0.09 (0.85)^c^	-	-0.65 (0.87)^d^	-	0.28 (0.69)^b^	-	0.45 (0.83)^a^	-	**0.23**

Scale − 2 to 2. N-L: Non-lethal tool, L: Lethal tool, C: Conservation-oriented intervention, TK: Tool kit. Means having the same superscript letter did not differ at *p* < 0.05 (ANOVA with Bonferroni correction). ^1^ = Tools included in conservation approach, ^2^ = Tools included in lethal approach. PCI_2_ values range from 0–1 and value closer to 1 indicate a maximum potential for conflict within the group^66^, i.e., high within group variation. Between-group differences reflecting a medium or large effect size (following guidelines proposed by Cohen^67^ in bold.

### Predictors of acceptance of management approaches

Bivariate correlations between WVOs, beliefs, emotions, acceptance of management approaches, and experiential variables are displayed in the Appendix Table 1A (where also the impact of stakeholder group (Partial eta^2^) on these variables is noted). In the first two steps of the regression model, Variance Inflation Factor (VIF) values were below 2.17 and given that the highest value was 3.63 only in the third step when control variables were added there is no cause for concern for multicollinearity regarding our variables of interest. In the first step, stronger mutualism orientation was positively associated with acceptance of the conservation approach and negatively associated with acceptance of the lethal approach, and the reverse was found for domination with an explained variance of 24% and 28%, respectively (Table 3). In the second step, the WVOs were still significant but with lower beta weights. Both beliefs and emotions were significant predictors of acceptance in the expected directions, but beliefs about ecosystem disservices did not significantly predict acceptance of the conservation approach (Table 3). In this step, the explained variance increased to 41% and 52%, respectively. In the third step, after adding experiential and control variables, the overall pattern of results remained, although beliefs about ES food/hunting were no longer a significant predictor of acceptance of the conservation approach (Table 3). The variables experience of hunting and stakeholder group were significant determinants of the acceptance of both approaches. In addition, age was negatively associated with acceptance of the conservation approach and men displayed higher acceptance of the lethal approach than women did. The explained variance increased significantly in the third step, but only with 3% and 1%, respectively.

**Table 3:** Determinants of acceptance of the conservation and the lethal approach, respectively, in three steps: (1) WVOs, (2) WVOs, beliefs, and emotions, (3) full model including control variables.

	Conservation approach				Lethal approach			
	B (SE)	β	95% CI	P value	B(SE)	β	95% CI	P value
Step 1.								
Mutualism	0.29(0.01)	0.33	(0.27,0.31)	0.001	− 0.27(0.01)	− 0.29	(-0.29,-0.25)	0.001
Domination	− 0.30(0.01)	− 0.28	(-0.33,-0.28)	0.001	0.42(0.01)	0.37	(0.40,0.45)	0.001
Step 2.								
Mutualism	0.13(0.01)	0.15	(0.11,0.15)	0.001	− 0.08(0.01)	− 0.08	(-0.10,-0.06)	0.001
Domination	− 0.14(0.01)	− 0.13	(-0.16,-0.11)	0.001	0.20(0.01)	0.17	(0.17,0.22)	0.001
ES food/hunting	− 0.04(0.01)	− 0.05	(-0.05,-0.02)	0.001	0.20(0.01)	0.26	(0.19,0.22)	0.001
ES nature	0.25(0.01)	0.27	(0.23,0.28)	0.001	− 0.17(0.01)	− 0.17	(-0.20,-0.15)	0.001
Ecosystem disservices	− 0.01(0.01)	− 0.01	(-0.03,0.02)	0.552	0.18(0.01)	0.18	(0.16,0.20)	0.001
Positive emotions	0.10(0.01)	0.17	(0.08,0.11)	0.001	− 0.10(0.01)	− 0.16	(-0.11,-0.08)	0.001
Negative emotions	− 0.14(0.01)	− 0.15	(-0.16,-0.12)	0.001	0.13(0.01)	0.13	(0.11,0.15)	0.001
Step 3.								
Mutualism	0.10(0.01)	0.11	(0.08,0.12)	0.001	− 0.05(0.01)	− 0.05	(-0.07,-0.03)	0.001
Domination	− 0.10(0.01)	− 0.09	(-0.12,-0.08)	0.001	0.16(0.01)	0.14	(0.14,0.18)	0.001
ES food/hunting	− 0.01(0.01)	− 0.01	(-0.02,0.01)	0.385	0.17(0.01)	0.22	(0.16,0.19)	0.001
ES nature	0.22(0.01)	0.23	(0.19,0.25)	0.001	− 0.14(0.01)	− 0.14	(-0.17,-0.12)	0.001
Ecosystem disservices	− 0.01(0.01)	− 0.01	(-0.03,0.01)	0.334	0.20(0.01)	0.19	(0.17,0.22)	0.001
Positive emotions	0.09(0.01)	0.16	(0.07,0.10)	0.001	− 0.09(0.01)	− 0.14	(-0.10,-0.07)	0.001
Negative emotions	− 0.11(0.01)	− 0.12	(-0.13,-0.09)	0.001	0.11(0.01)	0.11	(0.09,0.13)	0.001
Stakeholder group: The general public (D)	− 0.09(0.04)	− 0.03	(-0.17,-0.01)	0.021	0.18(0.04)	0.05	(0.10,0.25)	0.001
Stakeholder group: Farmers (D)	− 0.29(0.04)	− 0.10	(-0.37,-0.22)	0.001	0.33(0.04)	0.11	(0.25,0.40)	0.001
Stakeholder group: Hunters (D)	− 0.35(0.04)	− 0.16	(-0.43,-0.27)	0.001	0.12(0.04)	0.05	(0.04,0.20)	0.002
Visiting nature areas (D)	0.02(0.03)	0.01	(-0.04,0.07)	0.567	0.00(0.03)	0.00	(-0.06,0.05)	0.925
Birdwatching (D)	0.00(0.03)	0.00	(-0.06,0.05)	0.909	0.02(0.03)	0.01	(-0.03,0.08)	0.371
Hunting (D)	− 0.16(0.03)	− 0.08	(-0.22,-0.10)	0.001	0.24(0.03)	0.12	(0.18,0.30)	0.001
Gender (D)	− 0.02(0.02)	0.01	(-0.06,0.03)	0.474	− 0.08(0.02)	− 0.03	(-0.12,-0.04)	0.001
Age	0.00(0.00)	− 0.05	(0.00,0.00)	0.001	0.00(0.00)	0.01	(0.00,0.00)	0.239
Education (D)	− 0.02(0.02)	− 0.01	(-0.06,0.02)	0.261	0.03(0.02)	0.01	(-01,0.06)	0.140

D = dummy variable. ES = Ecosystem services. Birdwatchers were used as the reference group among stakeholders. Conservation approach: Step (1) Adj R^2^.24***, Step (2) Adj R^2^ 0.41*** (ΔR 0.17***), Step (3) Adj R^2^ 0.44*** (ΔR 0.03***) (*N* = 6020). Lethal approach: Step (1) Adj R^2^ 0.28***. Step (2) Adj R^2^ 0.51*** (ΔR 0.23***). Step (3) Adj R^2^ 0.54*** (ΔR 0.03***) (*N* = 6019). *** *p* < 0.001.

## Discussion

The present study examined stakeholder management acceptance as part of the social dimension of the social-ecological system. It provides important insights for the management of wildlife moving over large areas and where multiple levels of management are apparent. By using large samples and a theory that outlines how acceptance is linked to the social-ecological situation, through experiential processes, also integrating emotions with the cognitive hierarchy model, our study moreover contributes to the cumulative knowledge of stakeholders in wildlife management.

The emerging multi-level goose flyway management in Europe requires not only basic ecological knowledge and biological data concerning geese to establish a solid knowledge base of the ecological system, but also an understanding of the social dimensions where acceptance of management and beliefs about management and implementation are pertinent^6,17^. We found that despite an overall positive attitude towards geese, all groups acknowledge, to varying degree, the ecosystem disservices large numbers of geese may generate. Common problem perceptions have been revealed in previous research of e.g., non-native species^26^. Mutual perception of the problem among stakeholders is an important foundation to develop management, but platforms enabling stakeholder dialogue are still needed as part of the governance system^5^. This study showed that local level management has a key role to play in goose management, as all included stakeholder groups ascribe responsibility to and trust the local level. Birdwatchers and the general public displayed beliefs about a shared responsibility between the local, regional, and national levels, and also trusted these levels. Yet, our results from farmers and hunters reveal a gap between ascribed responsibility (higher) and trust (lower) at regional and national levels. Thus, there seems to be expectations on governance at the regional and national levels in these groups that are not met in practice, in other words, governance is not perceived to be implemented effectively (cf^48^). Given the importance of involving farmers and hunters in management on the ground, their low level of trust in the higher management levels is a significant concern for the emerging multi-level goose management system in the EU. As the multi-level governance system is relatively undeveloped, there is an untapped potential to establish a system, with the emphasis on the local level but with clear links for collaboration and communication between the levels.

By considering mean acceptance of management tools together with between-group and within-group differences, it is possible to identify tools that enjoy broad stakeholder acceptance (e.g., diversionary fields) and those that are not widely accepted (e.g., pricking of eggs). It is also possible to pinpoint tools that are accepted by some stakeholder groups and not others (e.g., derogation shooting when no permission is required), but also tools that are accepted by only some within a stakeholder group (e.g., auditory scaring). We found broad acceptance especially for diversionary fields, financial compensation for crop damage, visual scaring, the tool kit combining derogation shooting with scaring and diversionary fields, as well as for hunting during open hunting season. These results are in line with studies in other wildlife contexts showing high acceptance of non-lethal tools^14^. Still, consistent with studies of acceptance of hunting in Sweden^49^, hunting geese during open hunting season was accepted among the majority of stakeholders independent of interest. Financial compensation had the strongest support among birdwatchers and farmers. While this has also been identified as an accepted tool in other contexts e.g., seal management, concerns have been raised that it is a short-term tool, and if it is insufficient to cover costs or impedes on other than financial interests (e.g., recreation, cultural heritage) acceptance may be lower^50^. Tool kits can potentially gain broader stakeholder acceptance than stand-alone tools, as management must meet objectives for damage control and conservation. In line with expectations, our study shows an overall acceptance for the conservation approach across stakeholder groups, but acceptance for the lethal approach was mainly evident among hunters and farmers, not among birdwatchers, with the general public displaying an intermediate position. By examining acceptance for individual management tools as well as acceptance for general approaches, this study contributes to insights regarding the general strategies enjoying broad stakeholder acceptance, but also a more nuanced understanding (e.g., higher acceptance for visual versus auditory scaring). These insights can be used as part of the adaptive multi-level goose management system, complementing knowledge of what measures are effective in different contexts^51,52^ and data on flyways and goose behavior^53^. Since stakeholder acceptance of management is just one important issue, though, the use of tools with low stakeholder acceptance may be warranted under particular circumstances. In such situations, there is a need to carefully communicate why and how these tools are best used. Targeted collaboration involving dialogue, and respect for different perspectives may be required in cases of low acceptance in a specific stakeholder group^54,55^.

Our analyses support the determinants of management acceptance derived from our newly developed CEH model, including WVOs, beliefs about geese, and emotions geese evoke for acceptance of both the conservation approach and the lethal approach. In contrast to previous studies^13,14,38^, we provide support that the model-derived factors are relevant for, not only, acceptance of a lethal approach^20^, but also a conservation approach. Our data contradict that WVOs would be more predictive of acceptance of measures that are more harmful to wildlife^38^ but support the position of Jacobs et al.^20^ that WVOs are particularly important when there is a value conflict given the focus on tools that are more extreme at either side of the conservation – utilization continuum. In addition, analyses reveal that specific beliefs and emotions can explain variance in acceptance, thus adding to the explanatory value of WVOs. Furthermore, we confirm the relevance of experiential variables for acceptance as complementing to the higher-level cognitions (see Table 3, and Table 1A in the Appendix). Our study thereby suggests that in addition to value conflicts, also context-specific experiences, interpretations, and emotional reactions may drive acceptance of wildlife management. Our analyses further demonstrate that while acceptance of the lethal approach is boosted by beliefs about ecosystem disservices associated with geese, stakeholders may acknowledge these problems but still accept the conservation approach. While our study confirms that stakeholder group is a key variable associated with management acceptance, the explained variance was approximately doubled in analyses of determinants identified by drawing on the CEH model. Thus, the underlying rationale for stakeholder group differences can be found in WVOs, but also more specific beliefs and emotions partly emanating in specific experiences. This indicates that there is a need to address polarization in how stakeholders believe wildlife should be managed to avoid potential conservation conflicts. A better understanding of emotions towards wildlife has implications for wildlife management in practice for example by facilitating dialogue between managers and stakeholder groups^56^, and by supporting communication approaches that could mitigate feelings of fear among the public^57^. To further aid wildlife management, future research ought to focus on how new experiences of wildlife may lead to changes in beliefs, emotions, and acceptance.

There are limitations associated with this study worth consideration and additional research needed. Our data are restricted to a single country, but nonetheless covers diverse and relevant stakeholders in wildlife management. The large dominance of men in the samples (except the general public) was expected and mirrors stakeholder population characteristics, but an overall low representation of women is nevertheless a concern for wildlife management^5^. Methodologically, this study used the PCI_2_ as a measure of within-group differences and a visual tool to illustrate acceptance levels. The highest PCI_2_ was 0.38, indicating that the potential for conflict within stakeholder groups was relatively low. We found correlational support for the CEH model, but there is a need for experimental studies to corroborate it, and to further elaborate on e.g., how beliefs and emotions are linked, as well as to disentangle the importance of WVOs versus experiences as a basis for beliefs, emotions, and attitudes. Future research may examine interactions between levels (e.g., how WVOs interact with the emotions a particular species evokes). The links between attitudes and behaviors are further depicted in behavioral theories^58^ and a more extensive use of these theories to understand actions in wildlife management would likely be fruitful to deepen our understanding of stakeholder roles and engagement in these processes.

The empirical basis for exploring determinants of stakeholder acceptance in this study is unique, given the large samples and appropriate sample selection procedures, covering acceptance of lethal and non-lethal damage prevention tools, as well as conservation-oriented interventions, in a short time frame. Analyses of stakeholder acceptance of goose management have furthermore been relatively scarce thus far, why the study fills yet another knowledge gap. Management of goose populations is an illustrative example of the complexity and conflicts that managers need to handle for wildlife species worldwide. It involves populations moving over large areas, conservation of endangered species, harvest strategies of increasing species, and mitigation of ecosystem disservices, with many stakeholders at multiple management levels. Out study reveals that perceptions of problems are largely shared among stakeholders. It also shows widespread acceptance for the conservation of geese, but varying levels of acceptance for lethal tools and a gap between ascribed responsibility and trust in the higher management levels among hunters and farmers. Furthermore, our theoretical and empirical approach contributes to wildlife management research in more general terms by identifying and covering within- and between-stakeholder group differences^23,27^ as well as how acceptance is based both on general cognitions and experiences, via more specific cognitive and emotional processes. Similar stakeholder studies are needed to provide comprehensive guidance for the management of e.g., carnivores and ungulates in diverse socio-ecological contexts.

## Methods

### Study context

Geese are migratory waterbirds with long flyways straddling country borders. In Europe, some goose species are rare, e.g., the lesser white-fronted goose (*Anser erythropus*), while others, e.g., greylag goose (*Anser anser*) and barnacle goose (*Branta leucopsis*) have increased rapidly and reached superabundance^16^. Geese provide ecosystem services, such as game meat, seed dispersal, nutrient cycling stimulating plant productivity, and recreational experiences, but the large size of some populations also gives rise to disservices, including crop damage, bird strikes with aircrafts, detrimental effects on the habitats of other species, and contamination of beaches and parks^59,60^. An adaptive management approach focusing on systematic and stepwise learning from previous management outcomes has been adopted for waterbirds in North America^61,62^. Similarly, a multi-level adaptive management collaboration also including stakeholders (e.g., hunters, ornithologists, and farmers) was launched in Europe in 2015 (the European Goose Management Platform (EGMP) under the Agreement on the Conservation of African-Eurasian Migratory Waterbirds (AEWA))^17^. Goose management at all levels is governed by rules and regulations at the EU level, such as the Species and Habitats Directive and the Birds Directive. In Sweden, the goose management system includes governing bodies at the national and regional levels (the Swedish Environmental Protection Agency (SEPA), and the County Administrative Boards (CAB), respectively), but also arenas for collaboration at national and local level, respectively^5^. Goose management comprises damage prevention tools, including lethal tools (different forms of hunting), and non-lethal tools, such as scaring, as well as conservation-oriented interventions, such as financial compensation for crop damage, diversionary fields, sacrificial crops, and hunting free zones^63,64^.

### Sample

The study samples of the general public, hunters, and farmers were drawn from registers (the Swedish Population Register, the register of hunters at the SEPA, and the property register, respectively) using a simple random sampling approach. A commercial survey company conducted these data collections. Since there is no register of birdwatchers, all members of Birdlife Sweden were targeted for the birdwatcher study. A sufficiently large sample size to enable a multivariate analysis was used. Descriptions of the study populations and sampling approach respectively, can be found in Table 1. The samples deviated from their respective population in some regards (e.g., the general public sample displayed a higher education level, the sample of farmers were slightly older, more were women, and they owned larger farms, and the sample of hunters was slightly older). By controlling for demographic variables in the model testing these deviations, we find that they do not have a great impact on our main conclusions. Yet, they are relevant to consider e.g., when interpreting the descriptive results and when making comparisons with future studies. For additional information about the study samples see ^6,^^35^]

### Measures

Survey questions were developed amongst the interdisciplinary team of authors, drawing on previous research and operationalizations of theoretical concepts. Questions covered socio-demographic variables (gender, age, and education) and experiential variables measured frequency of engaging in outdoor activities (visiting natural areas, birdwatching, and hunting) on a five-point response scale (Experience = (1–4) every day or several times a week to one or a few times a year, No experience = (5) more seldom or never).

Evaluations of geese were assessed by means of a measure of *attitude* (i.e., a positive, neutral or negative evaluation of geese) and *acceptance capacity* (i.e., the population number acceptable to people)^35^. Two items were employed to assess attitude: “What do you think about having geese present in Sweden?” on a scale from 1 (I strongly dislike to have geese in Sweden) to 5 (I very much like to have geese in Sweden), and (b) “What is your attitude toward geese?” on a scale from 1 (Very negative) to 5 (Very positive) (α = 0.89). *Acceptance capacity* was also assessed by means of two items: “What is your perception of the goose population in your municipality?” on a scale from 1 (Far too few) to 5 (Far too many) and “What is your perception of whether the number of geese has changed the last 10 years in your municipality?” using a scale from 1 (Diminished a lot) to 5 (Increased a lot). The items were reverse coded so that a higher number reflected a higher acceptance capacity (α = 0.68). *Ascribed responsibility* was assessed by the question: “What responsibility do you believe that the following actors should have in the management of geese in Sweden?” (a) Local management groups with hunters, farmers, birdwatchers, and authorities, (b) The CAB at the regional level, (c) The SEPA at the national level, and (d) The EU at the European level on a response scale from 1 (No responsibility) to 5 (Very high responsibility), with the option to answer “don’t know”. *Trust* was assessed using the question “To what extent do you have trust in how the following actors handle goose management in Sweden?” at the same management levels as ascribed responsibility but with the response scale from 1 (Not at all) to 5 (To a great extent) and the option to answer “don’t know”. The “don’t know” answers were excluded from the analyses. *Acceptance of management* was assessed on a five-point scale coded in terms of a bipolar scale from − 2 (Very bad) to 2 (Very good) via an evaluation of a set of management tools for damage and conflict reduction, including lethal tools (L), non-lethal tools (N-L), conservation-oriented interventions (C), and tool kits (TK) (i.e. combinations of different tools) that are or may be used in goose management (short labels of tools are listed in Table 2 and full descriptions can be found in Appendix Table 2A).

*Mutualism and domination WVOs* were assessed by means of a Swedish translation of the short scale version proposed by Miller et al.^65^ but excluding one item from the domination scale (“Wildlife is on earth primarily for people’s benefit”). A response scale ranging from 1 (Totally disagree) to 5 (Totally agree) was used. *Beliefs about geese* reflected ecosystem services and disservices geese may provide. Respondents were asked: “To what extent do you believe the following to be a benefit [to cause problems] for humans or the ecosystem in Sweden?” with a response scale from 1 (Not at all) to 5 (To a great extent), also including the possibility to answer, “don’t know”. Before analyses, “don’t know” answers were excluded. The list of services and disservices is displayed in Appendix Table 3A. An exploratory factor analysis with varimax rotation was conducted after removing the “don’t know” answers, revealing three factors with eigenvalues > 1 (4.47, 1.88, 1.38), explaining 64% of the variance. The factors were labeled: Ecosystem disservices (6 items, including e.g., crop damage by geese, disease transmission from geese to humans), ecosystem service nature (ES nature) (4 items, including e.g., geese are beautiful to watch and contribute to biodiversity) and food/hunting (ES food/hunting) (2 items), including e.g., geese are good food). *Emotions geese evoke* were assessed using the question “To what extent do geese evoke the following emotions in you?” including a set of negative and positive emotions with a response scale from 0 (Not at all) to 6 (Very strong) (listed in Appendix Table 3A). Results from an exploratory factor analysis with varimax rotation disclosed two factors with eigenvalues of > 1 (5.20, 3.07) explaining 69% of the variance, positive and negative emotions, respectively. Means and standard deviations for WVOs, beliefs, and emotions for each stakeholder group are available in Appendix Table 3A and reliabilities for composite measures in Appendix Table 1A.

### Procedures

Data collection followed the ethical guidelines as stipulated in the 1964 Declaration of Helsinki. Prior to participating stakeholders were informed about the study and how personal information was handled. Before consenting to take part in the study participants were also instructed that participation was voluntary. Pseudonymization was conducted before data analyses to protect the privacy of participants. No ethical approval was needed since no sensitive personal information according to Swedish legislation (the Ethics Review Act 2003:460) was collected. The general public and farmers received the survey via postal mail, and two reminders were sent to those who did not respond. Birdwatchers received the survey via postal mail attached to their member magazine “Vår Fågelvärld”, with no reminders. Hunters were invited via postal letter to take part in the survey digitally. Subsequently, the survey was distributed to hunters via postal mail, including two postal reminders. Hunters with a publicly available phone number received an SMS reminder.

### Analyses

Analyses were conducted using SPSS 28. Initially, the samples from the four different stakeholder groups were compared with regards to socio-demographics and experience of outdoor activities using a univariate ANOVA in relation to age, and with Chi^2^ -tests for the remaining variables.

The first objective focusing on evaluations of geese and beliefs about the management system was analyzed by means of univariate ANOVAs with Bonferroni correction using Partial eta^2^ to assess effect size. The second objective involved analyses of acceptance of management including (a) acceptance level (mean values), (b) within-group differences by means of the Perceived Conflict Index (version PCI_2_ ; see^66^) ranging from 0 to 1, where a higher value denotes a higher potential for conflict within the group^23,50^, and (c) between-group differences utilizing univariate ANOVAs with Bonferroni correction and Partial eta^2^ to assess effect size. Analyses outlined management tools for which there is *overall acceptance* i.e., all group mean values are > 0.00. Tools with *low acceptance* are those for which all group mean values are ≤ 0.00. When group mean values included both positive and negative values, the tools were labelled *intermediate acceptance* when coupled with small between-group differences, but *disagreement* when group differences were medium or large. General management approaches were outlined by subjecting acceptance of management tools with medium to large between-group differences (according to guidelines proposed by Cohen^67^ i.e., partial eta^2^ > 0.06) to a confirmatory factor analysis.

To assess the third objective, determinants of acceptance for the two management approaches were tested using bivariate correlations (Pearson r) and hierarchical regression analyses. In addition, univariate ANOVAs with Bonferroni correction using Partial eta^2^ to assess effect size were utilized to assess the impact of stakeholder group on WVOs, beliefs, emotions, and acceptance. The model testing included a stepwise inclusion of WVOs (first), beliefs and emotions specifically associated with geese (second), experiential variables (frequency of involvement in outdoor activities) and control variables (including stakeholder group and socio-demographic factors) (third). This way the hierarchically ordered psychological variables were tested in step 1 and 2. In addition, we assessed the role of experiential variables as well as whether the pattern of results remained after including relevant controls.

## Electronic supplementary material

Below is the link to the electronic supplementary material.

Supplementary Material 1

## Data Availability

The datasets generated and analysed during the current study are available from the corresponding author on reasonable request.
