# Supplementary material for: Novel insights into the cognitive, emotional, and experiential dimensions of stakeholder acceptance of wildlife management
Source: Sci Rep. 2024 Nov 27;14:29479. doi: 10.1038/s41598-024-80661-2 (PMC11603312; doi:10.1038/s41598-024-80661-2)
Supplement: Supplementary file 1 — Supplementary Material 1 [file 41598_2024_80661_MOESM1_ESM.docx]

**Appendix**

1. **LETHAL TOOLS**


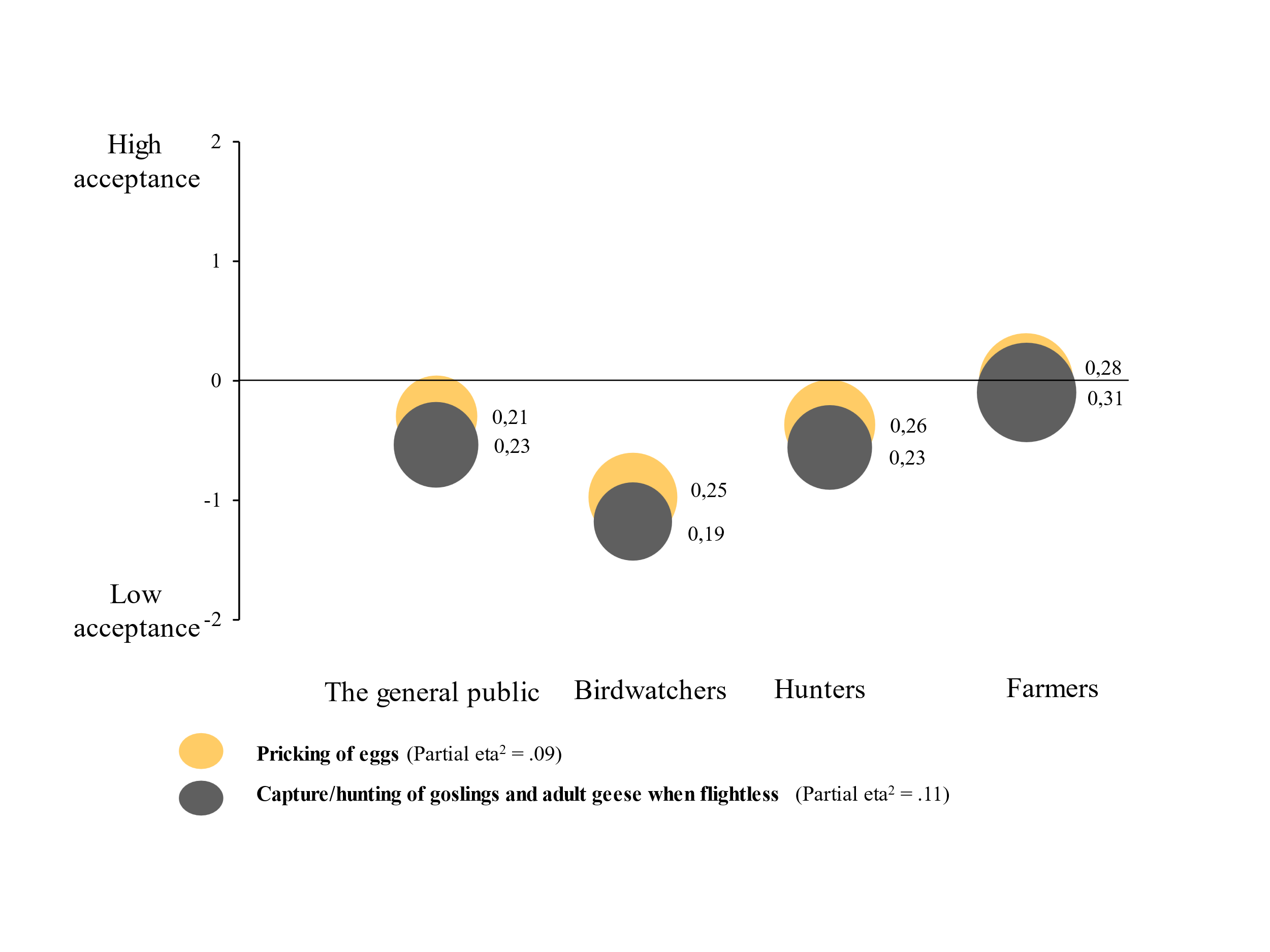


1. **NON-LETHAL TOOLS**


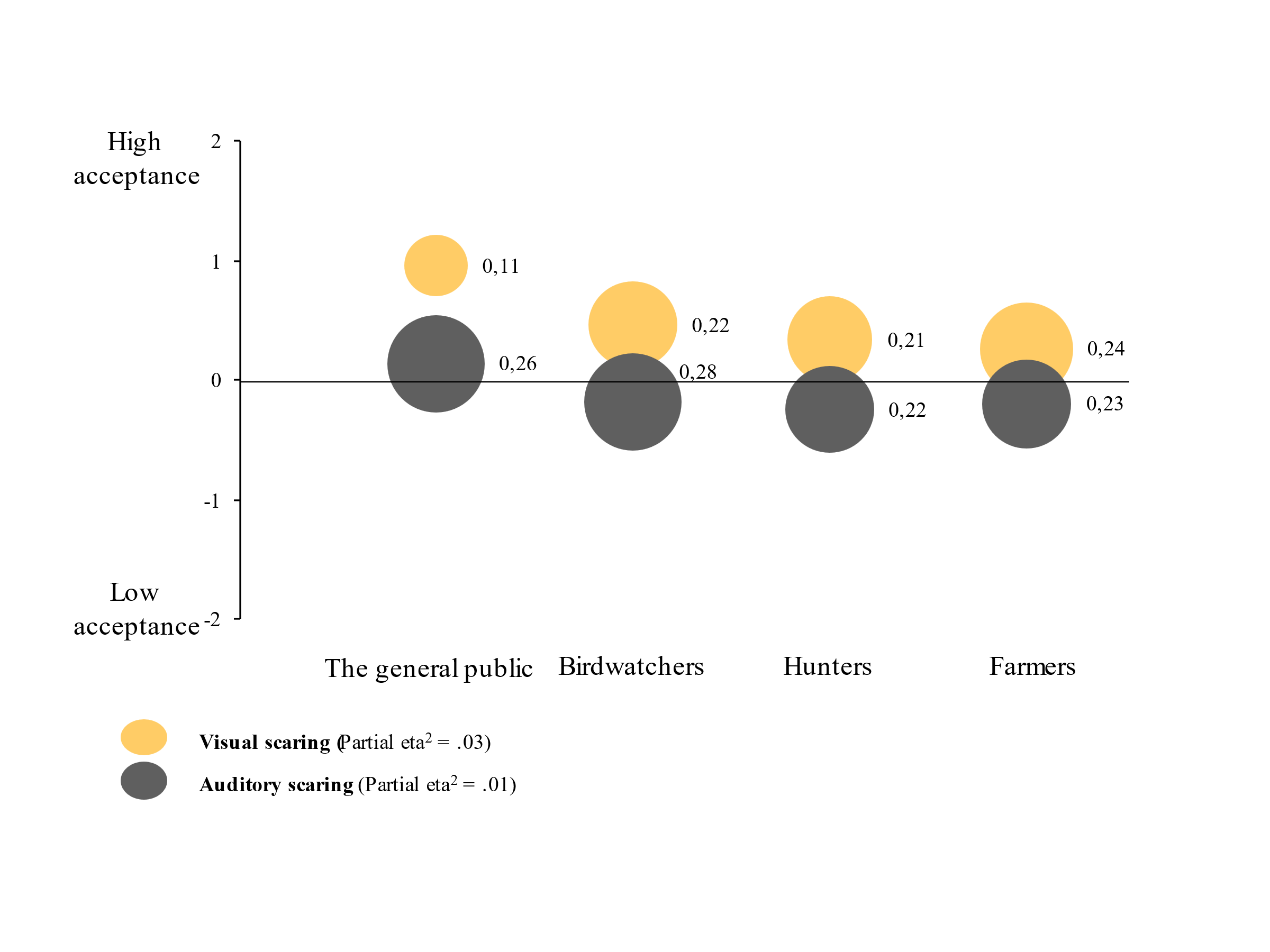


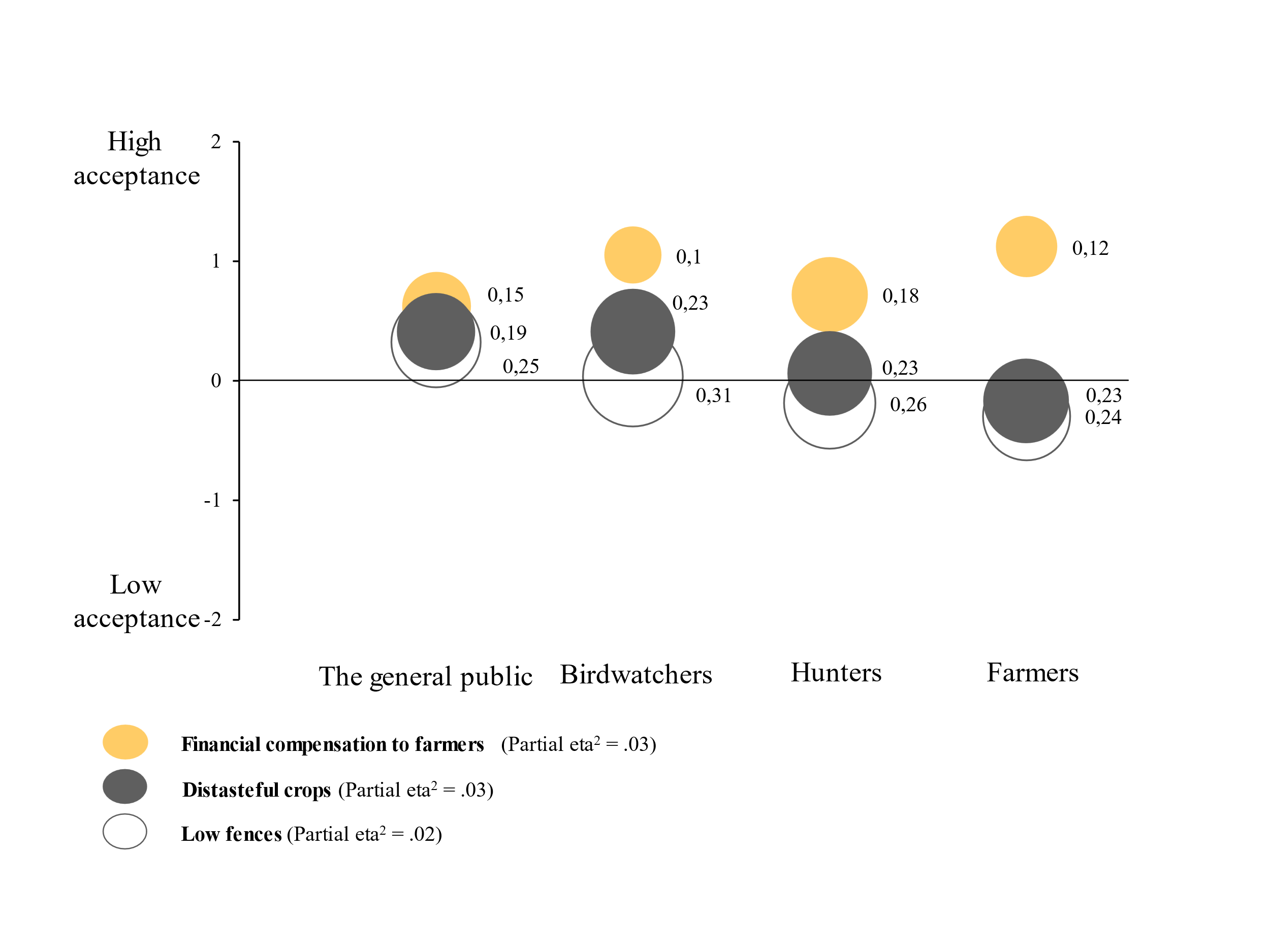


1. **CONSERVATION-ORIENTED TOOLS**


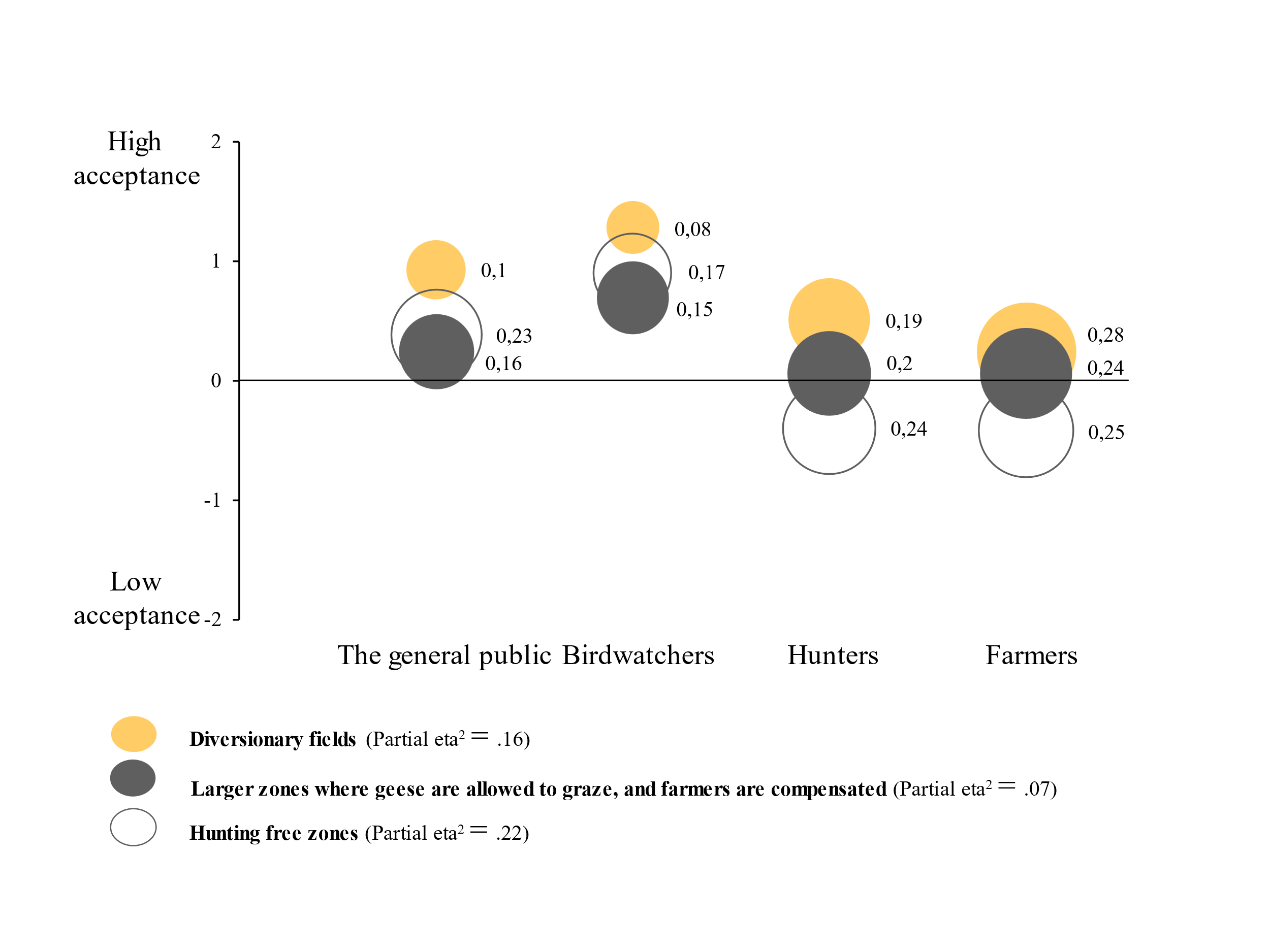


1. **TOOL KITS**


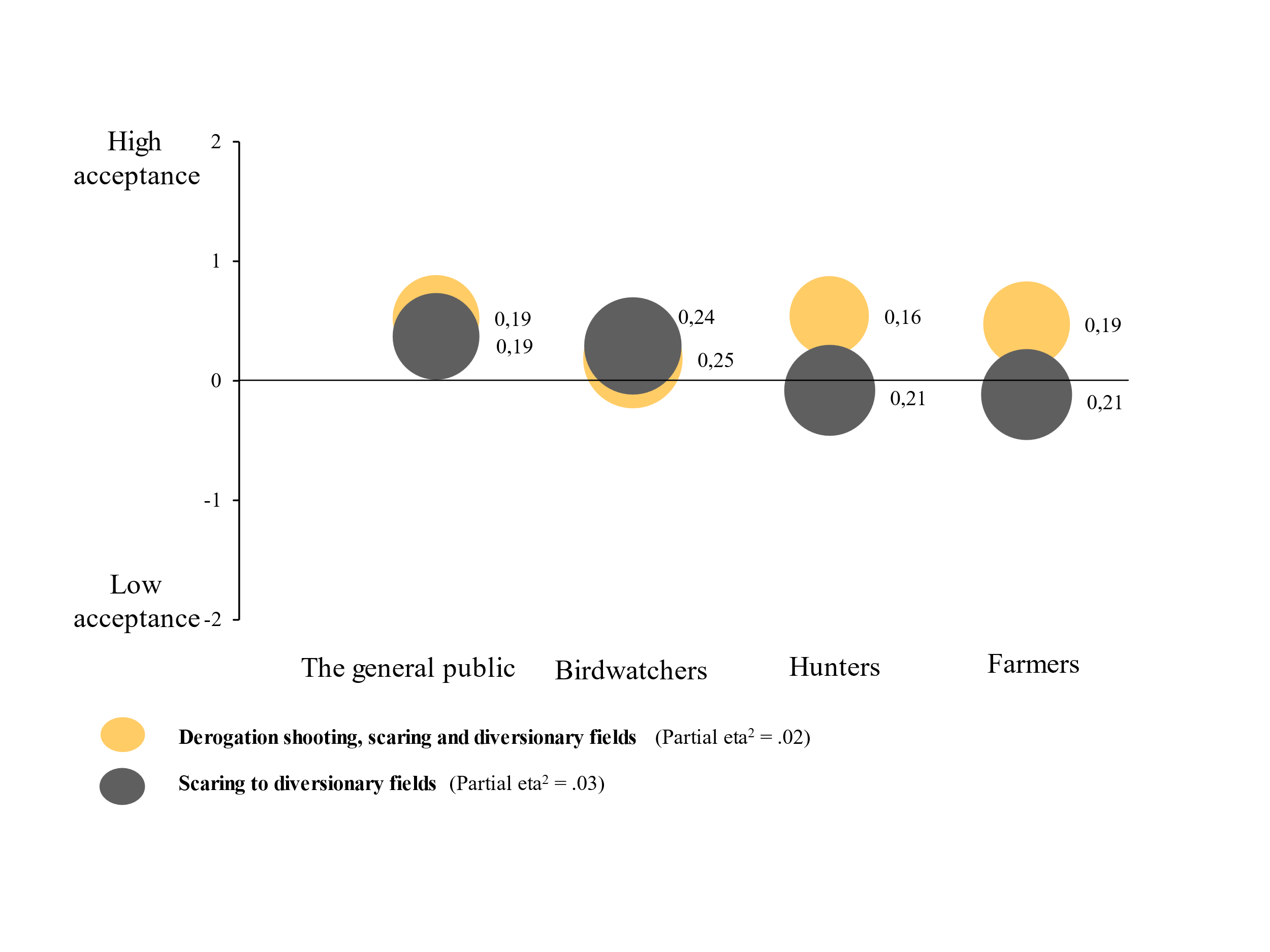


Figure 1A. Acceptance for A) Lethal tools (hunting during open hunting season and derogation shooting can be found in Figure 3), B) Non-lethal tools (top panel: Scaring: bottom panel: Remaining tools), C) Conservation-oriented tools, and D) Tool kits.

Table 1A. Bivariate correlations for wildlife value orientations, beliefs, emotions, acceptance, and experiential variables.

|  | Wildlife value orientation | | Specific beliefs | | | Emotions | | Acceptance | | Experiential variables | |
| --- | --- | --- | --- | --- | --- | --- | --- | --- | --- | --- | --- |
|  | Mutualism | Domination | ES food/  hunting | ES nature | Ecosystem disservices | Positive | Negative | Acc Pres | Acc Lethal | Nature areas | Bird-watching |
| Mutualism ^a^ | α = .81 |  |  |  |  |  |  |  |  |  |  |
| Domination ^a^ | -.27*** | α = .67 |  |  |  |  |  |  |  |  |  |
| ES food/ hunting ^a^ | -.21*** | .17*** | α = .70 |  |  |  |  |  |  |  |  |
| ES nature ^a^ | .43*** | -.33*** | -.03** | α = .85 |  |  |  |  |  |  |  |
| Ecosystem disservices ^a^ | -.18*** | .31*** | .20*** | -.38*** | α = .84 |  |  |  |  |  |  |
| Positive emotions ^b^ | .43*** | -.28*** | -.11*** | .63*** | -.39*** | α = .92 |  |  |  |  |  |
| Negative emotions ^b^ | -.20*** | .33*** | .06*** | -.46*** | .39*** | -.26*** | α = .90 |  |  |  |  |
| Acc Pres ^a^ | .40*** | -.35*** | -.14*** | .54*** | -.30*** | .48*** | -.39*** | α = .69 |  |  |  |
| Acc Lethal ^a^ | -.37*** | .43*** | .37*** | -.49*** | .47*** | -.48*** | .41*** | -.44*** | α = .72 |  |  |
| Nature areas (D) | .11*** | -.22*** | .00 | .20*** | -.16*** | .23*** | -.19*** | .18*** | -.19*** |  |  |
| Bird-watching (D) | .27*** | -.31*** | -.13*** | .33*** | -.20*** | .40*** | -.29*** | .33*** | -.34*** | .36*** |  |
| Hunting (D) | -.32*** | .24*** | .27*** | -.28*** | .14*** | -.21*** | .20*** | -.36*** | .37*** | -.03** | -.25*** |

*** p < 0.001, ** p < 0.01, *** p < .05. a) Scale 1-5, b) Scale 0-6, D = Dummy. ES = Ecosystem services, Acc Pres = Acceptance Preservation approach, Acc Lethal = Acceptance lethal approach. Partial eta^2^ for the effect of stakeholder group on: Mutualism = .15, Domination .16, ES food/hunting .08, ES nature .19, EDS .05, Positive emotions .21, Negative emotions .13, Acc Pres .22, Acc Lethal .23.

Table 2A. Tools used in goose management.

|  | Short labels |
| --- | --- |
| **Lethal tools for damage and conflict reduction** |  |
| Hunting during open hunting season (certain time of the year) | Hunting during open hunting season |
| Derogation shooting to prevent damages only after permission by the County Administrative Board. | Derogation shooting under permission |
| Derogation shooting to prevent damages by own initiative (no permission required) | Derogation shooting no permission |
| Capture/hunting of goslings and adult birds during molt and when they are flightless during breeding | Capture/hunting of goslings and adult geese when flightless |
| Pricking of eggs | Pricking of eggs |
| **Non-lethal tools for damage and conflict reduction** |  |
| Economic compensation to farmers affected by damage caused by geese | Economic compensation |
| Visual scaring measures (e.g., flags and kites) in fields | Visual scaring |
| Auditory scaring measures (sound) in fields (e.g., propane cannons) | Auditory scaring |
| Use crops/grass that are distasteful in places where you do not want geese present (e.g., fields with growing crops and parks) | Distasteful crops |
| Low fences at beaches/shore pastures to keep geese out | Low fences |
| **Conservation-oriented interventions for damage and conflict reduction** |  |
| Diversionary fields, i.e., fields where geese are attracted by attractive crops/feeding and can graze undisturbed (‘sacrificial crop’) | Derivation fields |
| Larger zones /areas where landowners are compensated according to a scheme to allow geese to graze on their land | Larger zones where geese are allowed to graze and farmers are compensated |
| Hunting free zones to ensure that geese have a refuge | Hunting free zones |
| **Tool kits for damage and conflict reduction** |  |
| Combine derogation shooting with scaring measures and diversionary fields. | Derogation shooting, scaring and diversionary fields |
| Scaring visually or by sound to diversionary fields where they can graze undisturbed | Scaring geese to diversionary fields |

Table 3A. Means and standard deviations for wildlife value orientations, beliefs about ecosystem services and disservices, and positive and negative emotions.

|  | The general public | Birdwatchers | Hunters | Farmers |
| --- | --- | --- | --- | --- |
|  | M (SD) | M (SD) | M (SD) | M (SD) |
| **Wildlife value orientations^1^** |  |  |  |  |
| Mutualism | 3.35 (0.97)^b^ | 3.76 (0.95)^a^ | 2.87 (0.99)^c^ | 2.86 (0.97)^c^ |
| Domination | 2.27 (0.91)^b^ | 1.69 (0.69)^c^ | 2.30 (0.85)^b^ | 3.26 (1.14)^a^ |
| **Ecosystem services (ES)^2^** |  |  |  |  |
| Geese are beautiful to watch†† | 3.55 (1.05)^b^ | 4.22 (0.86)^a^ | 3.31 (1.07)^c^ | 3.15 (1.17)^d^ |
| Geese contribute to increased nature tourism/eco-tourism†† | 2.84 (1.23)^b^ | 3.10 (1.14)^a^ | 2.40 (1.10)^c^ | 2.06 (1.06)^d^ |
| Geese contribute to higher biodiversity†† | 3.26 (1.06)^b^ | 3.80 (1.00)^a^ | 3.08 (1.08)^c^ | 2.62 (1.13)^d^ |
| Geese are an important part of the ecosystem (e.g., graze to keep shore meadows open)†† | 3.49 (1.16)^b^ | 3.97 (0.97)^a^ | 3.11 (1.13)^c^ | 2.65 (1.21)^d^ |
| Goose hunting is appreciated† | 3.11 (1.32)^b^ | 2.44 (1.23)^c^ | 3.60 (1.10)^a^ | 3.23 (1.26)^b^ |
| Geese are good food† | 3.13 (1.41)^b^ | 3.04 (1.36)^b^ | 3.55 (1.20)^a^ | 3.11 (1.31)^b^ |
| Ecosystem services food/hunting† | 3.12 (1.24)^b^ | 2.73 (1.24)^c^ | 3.57 (1.01)^a^ | 3.17 (1.16)^b^ |
| Ecosystem services nature†† | 3.32 (0.96)^b^ | 3.81 (0.80)^a^ | 3.03 (0.90)^c^ | 2.71 (1.00)^d^ |
| **Ecosystem disservices (EDS)^2^** |  |  |  |  |
| Crop damage by geese in agriculture | 3.85 (1.12)^c^ | 3.51 (0.94)^d^ | 4.02 (0.94)^b^ | 4.21 (0.93)^a^ |
| Angry geese on beaches and in parks | 2.85 (1.33)^a^ | 2.52 (1.23)^b^ | 2.88 (1.31)^a^ | 2.74 (1.35)^a^ |
| Risk for collisions at airports | 3.35 (1.19)^b^ | 3.03 (1.05)^c^ | 3.25 (1.12)^b^ | 3.59 (1.16)^a^ |
| Disease transmission from geese to humans | 2.66 (1.23)^b^ | 2.07 (1.01)^c^ | 2.61 (1.14)^b^ | 3.02 (1.30)^a^ |
| Droppings in parks, on beaches and golf courses | 3.70 (1.28)^ab^ | 3.33 (1.23)^c^ | 3.79 (1.18)^a^ | 3.62 (1.29)^b^ |
| Over fertilization of water courses caused by geese | 3.26 (1.27)^a^ | 2.67 (1.16)^b^ | 3.19 (1.19)^a^ | 3.11 (1.29)^a^ |
| Ecosystem disservices | 3.32 (1.01)^b^ | 2.95 (0.91)^c^ | 3.37 (0.87)^ab^ | 3.47 (0.97)^a^ |
| **Positive emotions^3^** |  |  |  |  |
| Relief | 0.87 (1.45)^b^ | 1.42 (1.80)^a^ | 1.03 (1.31)^b^ | 0.55 (1.11)^c^ |
| Enthusiasm | 1.37 (1.70)^c^ | 3.09 (1.91)^a^ | 1.75 (1.67)^b^ | 0.93 (1.39)^d^ |
| Pleasure | 1.87 (1.85)^b^ | 3.25 (1.81)^a^ | 1.99 (1.70)^b^ | 1.23 (1.58)^c^ |
| Interest | 1.96 (1.77)^c^ | 2.55 (1.71)^b^ | 3.97 (1.58)^a^ | 1.49 (1.64)^d^ |
| Joy | 2.11 (1.88)^b^ | 3.75 (1.70)^a^ | 2.29 (1.72)^b^ | 1.37 (1.59)^c^ |
| Positive emotions | 1.66 (1.52)^c^ | 3.11 (1.50)^a^ | 1.94 (1.38)^b^ | 1.13 (1.25)^d^ |
| **Negative emotions^3^** |  |  |  |  |
| Sadness | 0.37 (1.06)^b^ | 0.15 (0.64)^c^ | 0.62 (0.96)^a^ | 0.71 (1.56)^a^ |
| Despair | 0.55 (1.29)^c^ | 0.19 (0.74)^d^ | 0.82 (1.22)^b^ | 1.27 (2.00)^a^ |
| Worry | 0.68 (1.30)^b^ | 0.24 (0.79)^c^ | 0.72 (1.09)^b^ | 1.06 (1.80)^a^ |
| Disgust | 0.76 (1.50)^c^ | 0.27 (0.87)^d^ | 0.97 (1.32)^b^ | 1.19 (1.87)^a^ |
| Anger | 0.79 (1.48)^c^ | 0.31 (0.91)^d^ | 1.10 (1.46)^b^ | 1.51 (2.07)^a^ |
| Fear | 0.85 (1.41)^a^ | 0.12 (0.49)^d^ | 0.59 (0.89)^b^ | 0.46 (1.13)^c^ |
| Irritation | 1.35 (1.78)c | 0.71 (1.28)^d^ | 1.71 (1.70)^b^ | 2.13 (2.21)^a^ |
| Negative emotions | 0.77 (1.13)^c^ | 0.29 (0.61)^d^ | 0.94 (0.99)^b^ | 1.24 (1.57)^a^ |

Note. ^1^ Scale 1-5. ^2^ Scale 1-5. ^3^ Scale 0-6. † Ecosystem services food/hunting. †† Ecosystem services nature. Means having the same superscript letter did not differ at p < 0.05 (ANOVA with Bonferroni correction).
